# Saving life and limb: limb salvage using external fixation, a multi-centre review of orthopaedic surgical activities in Médecins Sans Frontières

**DOI:** 10.1007/s00264-014-2451-6

**Published:** 2014-07-20

**Authors:** Marie Jeanne Bertol, Rafael Van den Bergh, Miguel Trelles Centurion, Hyacinthe Kenslor Ralph D, Jean-Paul Basimuoneye Kahutsi, Abdul Qayeum Qasemy, Jacky Jean, Alberta Majuste, Theophile Kubuya Hangi, Samsoor Safi

**Affiliations:** 1 Operational Centre Brussels, Médecins Sans Frontières, Rue Dupré 94, Brussels, 1090 Belgium; 2 Emergency Surgical/Trauma Referral Centre “Nap Kembe”, Médecins Sans Frontières – Haiti, Tabarre Port-au-Prince, Haiti; 3 Referral General Hospital of Masisi, Médecins Sans Frontières – Democratic Republic of the Congo, Nord Kivu Province, Democratic Republic of the Congo; 4 Kunduz Trauma Centre, Médecins Sans Frontières – Afghanistan, Kunduz, Afghanistan

**Keywords:** External fixation, Natural disasters, Limb salvage, Amputation, Orthopaedics, Operational research

## Abstract

**Purpose:**

While the orthopaedic management of open fractures has been well-documented in developed settings, limited evidence exists on the surgical outcomes of open fractures in terms of limb salvage in low- and middle-income countries. We therefore reviewed the Médecins Sans Frontières-Operational Centre Brussels (MSF-OCB) orthopaedic surgical activities in the aftermath of the 2010 Haiti earthquake and in three non-emergency projects to assess the limb salvage rates in humanitarian contexts in relation to surgical staff skills.

**Methods:**

This was a descriptive retrospective cohort study conducted in the MSF-OCB surgical programmes in the Democratic Republic of Congo (DRC), Afghanistan, and Haiti. Routine programme data on surgical procedures were aggregated and analysed through summary statistics.

**Results:**

In the emergency post-earthquake response in Haiti, 81 % of open fracture cases were treated by amputation. In a non-emergency project in a conflict setting in DRC, relying on non-specialist surgeons receiving on-site supervision and training by experienced orthopaedic surgeons, amputation rates among open fractures decreased by 100 to 21 % over seven years of operations. In two trauma centres in Afghanistan (national surgical staff supported from the outset by expatriate orthopaedic surgeons) and Haiti (national musculoskeletal surgeons trained in external fixation), amputation rates among long bone open fracture cases were stable at 20 % and <10 %, respectively.

**Conclusions:**

Introduction of and training on the proper use of external fixators reduced the amputation rate for open fractures and consequently increased the limb salvage rates in humanitarian contexts where surgical care was provided.

## Introduction

Injury remains a leading cause of death worldwide [1], and deaths from trauma—including injuries from natural and human disasters—are projected to rise significantly until 2020 [2]. Many cases of trauma are amenable to surgical intervention; however, the vast majority of disasters and conflicts occur in low- and middle-income countries (LMICs) [3, 4] where there is a shortage of surgical capacity [5].

Surgical treatment of open fractures has been extensively documented in the literature, and the management principles of such conditions are well-established among the orthopaedic community [6, 7]. Emergency debridement and copious irrigation have been the hallmark of treatment of open fractures, and the use of external fixation to decrease the incidence of infection has been recommended since Gustilo and Anderson’s historic study in 1976 [8]. In cases with mangled extremities, the decision for limb salvage versus amputation is guided by several factors, including the prognosed recovery following salvage or amputation [9, 10]. However, under programme/field conditions in many LMICs, the functional outcome of amputation is in no way comparable to the ideal clinical setting, given that resources for prosthetics are usually not readily available, and amputation is thus more likely to result in lifelong disability.

Médecins Sans Frontières (MSF) is an international, independent, medical humanitarian organization, which has delivered emergency aid, including surgical care, for over 40 years in more than 70 countries [11]. In MSF surgical projects, the decision to amputate a limb is a very difficult one [12–19]. It depends on the surgical skills of the available human resources and the available assets of the health facility, while taking into consideration social and religious factors [20]. Damage-control orthopaedics has re-introduced the use of external fixators for the treatment of open fractures in disaster settings, both natural and man-made, where resources are channelled to treating as many patients as possible while giving the best possible management to each one [21]. In MSF projects, external fixations are performed by orthopaedic surgeons, as well as by trained general surgeons and medical doctors with surgical skills.

The purpose of this study was to review the orthopaedic surgical activities performed during the first ten weeks after the Haiti earthquake of 2010, and in three ongoing MSF-Operational Centre Brussels (MSF-OCB) projects relying on surgical staff with differing technical skills, in order to assess the limb salvage rates in humanitarian contexts under programme conditions and in relation to surgical staff skills.

## Methods

### Study design

This was a descriptive retrospective cohort study utilizing routine programmatic data from MSF surgical programmes in the Democratic Republic of Congo (DRC), Afghanistan, and Haiti.

### Study setting and period

The study was conducted in the surgical programmes of the emergency intervention following the 2010 earthquake in Haiti (in Chancerelle, Cité-Soleil and Sarthe hospitals), and in the programmes of the non-emergency MSF-OCB projects in Masisi General Referral Hospital (DRC), Kunduz Trauma Centre (Afghanistan), and Tabarre Trauma Centre (Haiti).

On January 12th, 2010, a magnitude 7.0 earthquake devastated Port-au-Prince in Haiti, taking 200,000 to 300,000 lives and injuring another 300,000. More than one million individuals were displaced, living in tents with very little access to healthcare. As MSF-OCB had been operational in Haiti for 19 years prior to the earthquake, a rapid, well-informed response was mounted, with the teams on the ground providing the initial base for medical action. A focus was placed on providing surgical care in three health structures, Chancerelle, Cité-Soleil and Sarthe hospitals. In this study, data from January 16th to March 21st, 2010 are presented.

The General Referral Hospital of Masisi in Nord Kivu province, DRC, has been supported by MSF-OCB since September 2007. Masisi is a zone of open conflict between the national forces of the DRC and various armed militias. MSF-OCB collaborates with the Ministry of Health (Ministère de la Santé Publique) in offering free access to general health care, including provision of surgical care at the Masisi hospital, and support to surgical interventions in two outlying health centres. All surgical activities from September 2007 to June 2013 were included in this study.

Over the course of 2012–2013, MSF-OCB managed two trauma centres: Kunduz Trauma Centre in Afghanistan and Tabarre Trauma Centre in Haiti. Kunduz Trauma Centre in the north-eastern part of Afghanistan was opened in August 2011. The province has been impacted severely by the ongoing conflict in Afghanistan, in particular following the 2010 United States troop surge. The MSF hospital in Kunduz fills a specific gap in trauma care in the region: before the presence of MSF, only two public hospitals in the province were able to receive war wounded, while the private sector does not offer care for trauma patients. Data from Kunduz from January 2012 to June 2013 were analysed. The Tabarre Trauma Centre in Port-au-Prince, Haiti was opened by MSF-OCB in February 2012 and provides specialized care for trauma patients, including visceral surgery and orthopaedic care. It was created to address the gap in trauma care in the aftermath of the 2010 earthquake, and accepts all types of trauma patients. All surgical data from February 13th 2012 to June 30th, 2013 are presented in this study.

### External fixation procedures

In MSF surgical projects, all open fractures (from Gustilo Type I to III) are treated with prophylactic antibiotic therapy, an emergency debridement with copious irrigation followed by immobilization, be it with a circular cast with a window for subsequent wound care or by external fixation. All MSF surgical projects are provided with a standard Gexfix (Carouge, Switzerland) external fixation kit, which is generally used as definitive treatment for long bone open fractures in adults and children, as well as for open pelvis injuries. In the field, expatriate orthopaedic and general surgeons instruct, train and supervise national surgeons and doctors with surgical skills on the proper techniques and approaches in the application of the external fixation system.

### Data collection

All surgical procedures performed were routinely collected using a standardized logbook and electronic database (Microsoft Excel) developed for institutional operational needs. These data were aggregated at MSF-OCB headquarters and reviewed for completeness and accuracy and verified against the patient records. Interventions and their characteristics were classified according to a standardized system described previously [22].

### Ethics considerations

The study satisfied the Médecins Sans Frontières Ethics Review Board (Geneva, Switzerland) criteria for studies using routinely collected data and was approved by the *Comité National de Bioéthique* of the *Ministère de la Santé Publique et de la Population* of Haiti. The study was conducted as a retrospective analysis of routine programme data, and informed consent was thus not sought from study subjects; however, identifying information was removed from all patient records prior to analysis.

## Results

### Haiti post-earthquake emergency intervention

**Fig. 1:**
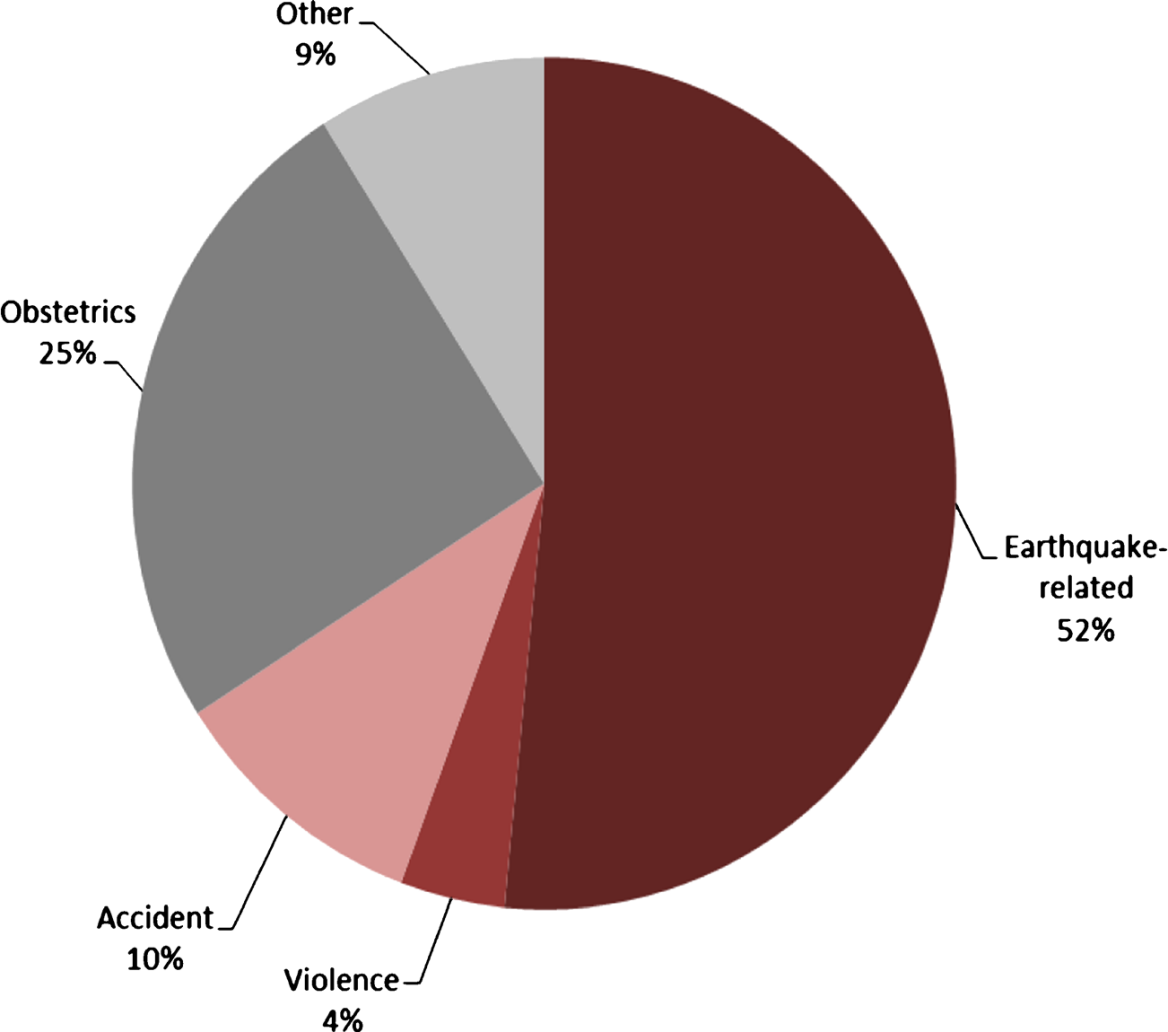
Indications of surgical intervention (new cases) in the post-earthquake emergency intervention, Port-au-Prince, Haiti, 16th January to 21st March 2010

**Fig. 2:**
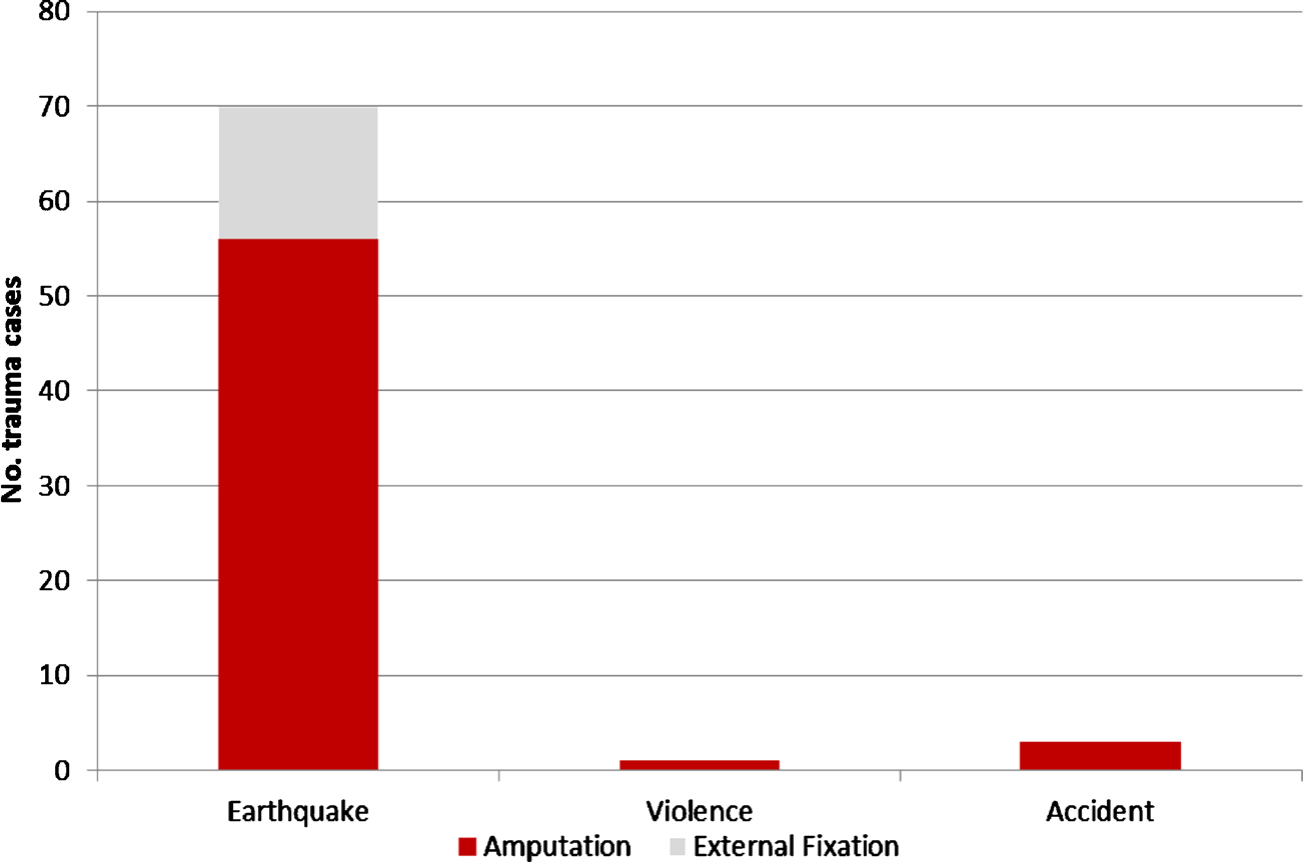
Surgical interventions in open fractures by nature of injury in the post-earthquake emergency intervention, Port-au-Prince, Haiti, 16th January to 21st March 2010

Following the earthquake in Port-au-Prince, Haiti, on January 12th 2010, the three hospitals of MSF-OCB saw a total of 778 new surgical cases over the first ten weeks of the emergency response, half of whom had injuries directly related to the earthquake (Fig. 1). During this response, 1,309 surgical interventions were carried out on these cases, with the majority (852; 65 %) for immediate debridement and follow-up wound care. Out of the 778 cases, 74 cases with open fractures were seen; the majority of these (60; 81 %) were treated by amputation (Fig. 2). Most amputations (34; 49 %) were conducted in the week after the earthquake; from the second week, a steady decline in amputations was noted from nine to 1.5 per week.

### Orthopaedic surgery in the General Referral Hospital Masisi, DRC

**Fig. 3:**
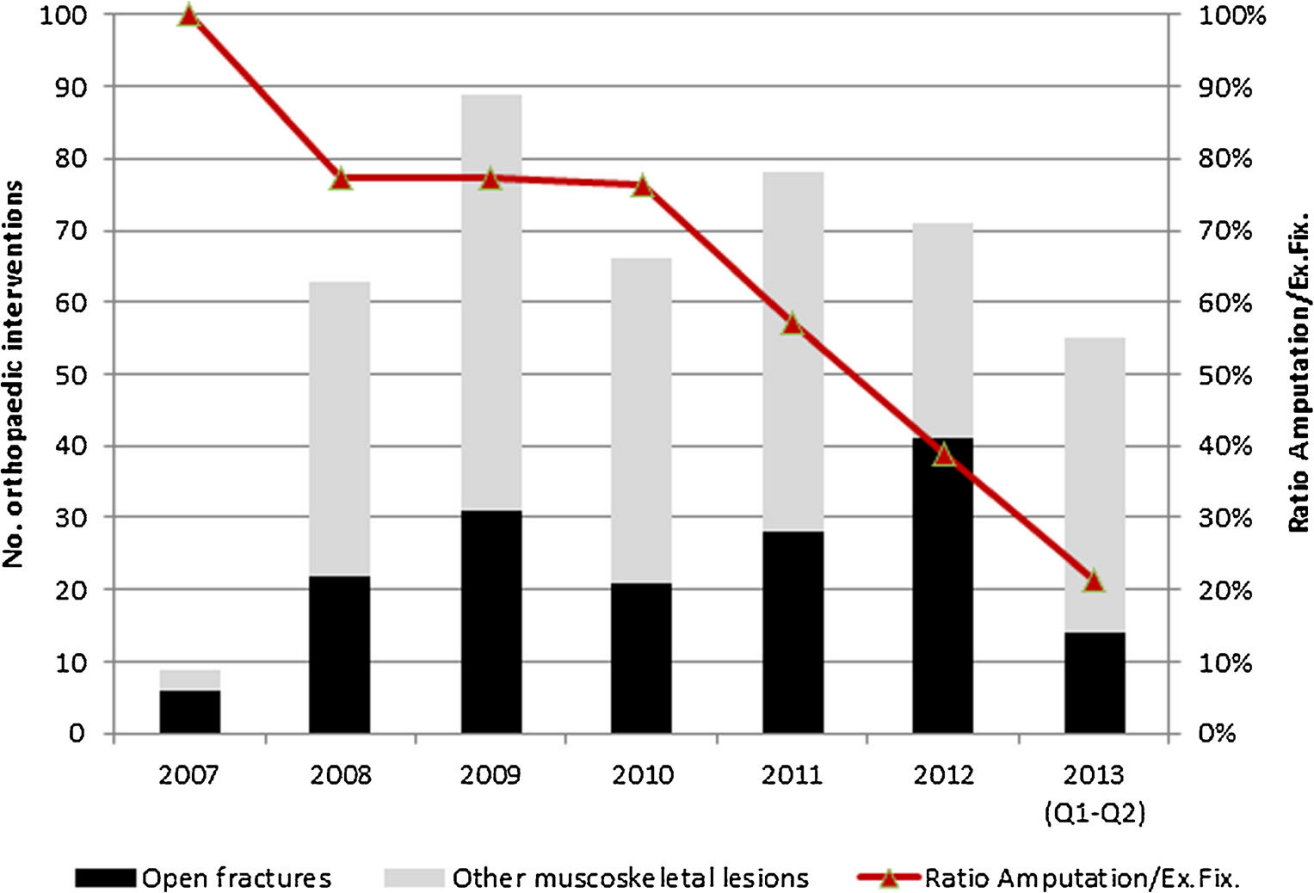
Orthopaedic interventions and ratio of amputation versus external fixation among open fracture cases at the Masisi General Referral Hospital, DRC, 2007–2013

Over the study period, 431 orthopaedic interventions were documented for various pathologies. These included amputations, osseous curettage for osteomyelitis, application of external fixators, closed reduction and casting, tenorrhaphies and tractions. Of these 431 interventions, 163 were performed for open fractures. The amputation rate declined steadily from 100 % in 2007 to 39 % in 2012 and 21 % during the first half of 2013 (Fig. 3). The number of interventions for external fixation remained the same from 2008 to 2013 while the rate of close reduction and application of cast has steadily increased from 2009 to 2013.

### Orthopaedic surgery in MSF-OCB trauma centres, Afghanistan and Haiti

**Fig. 4:**
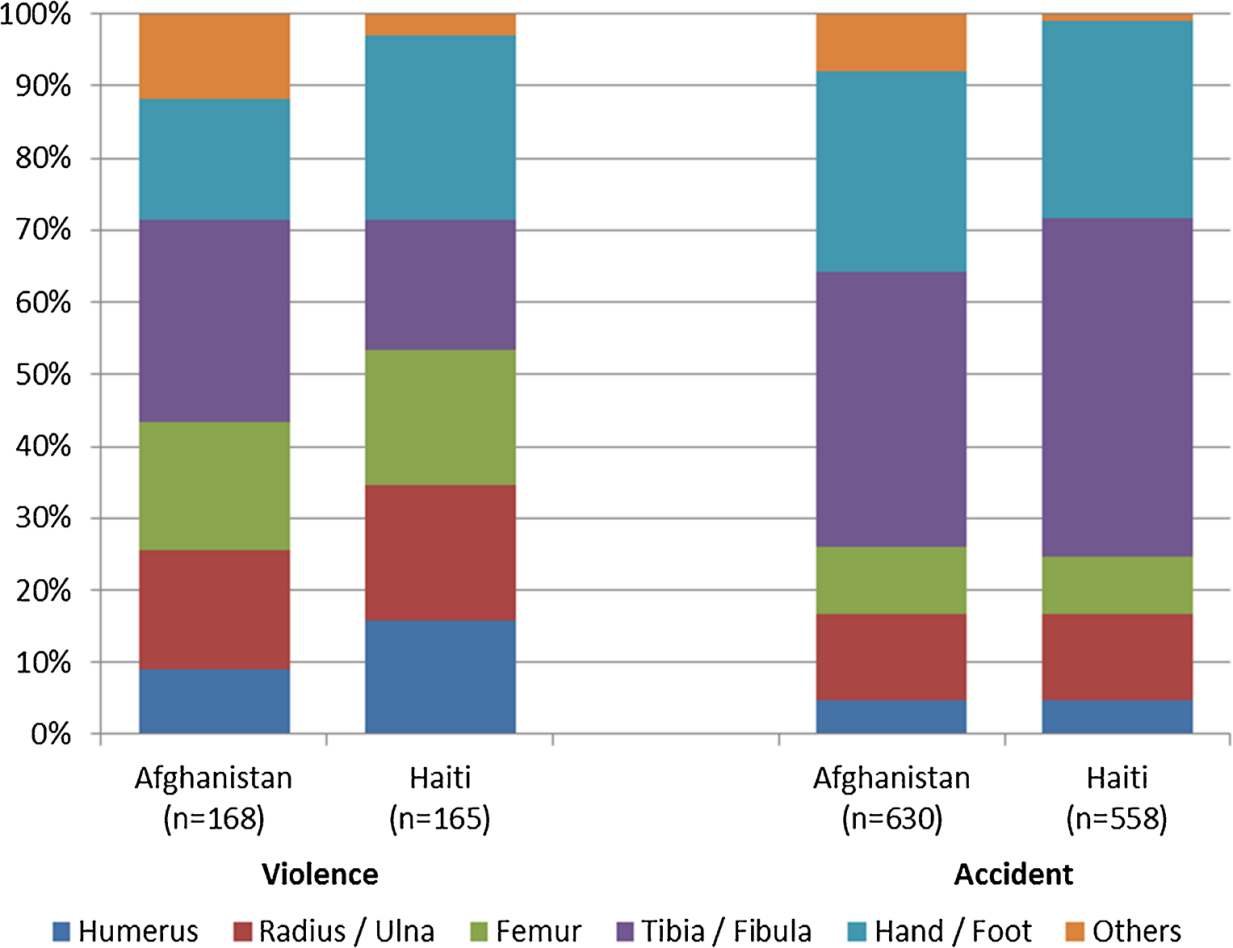
Skeletal region of open fractures by origin of trauma in the MSF-OCB trauma centres of Kunduz, Afghanistan and Tabarre, Haiti, 2012–2013 (2nd quarter)

**Fig. 5:**
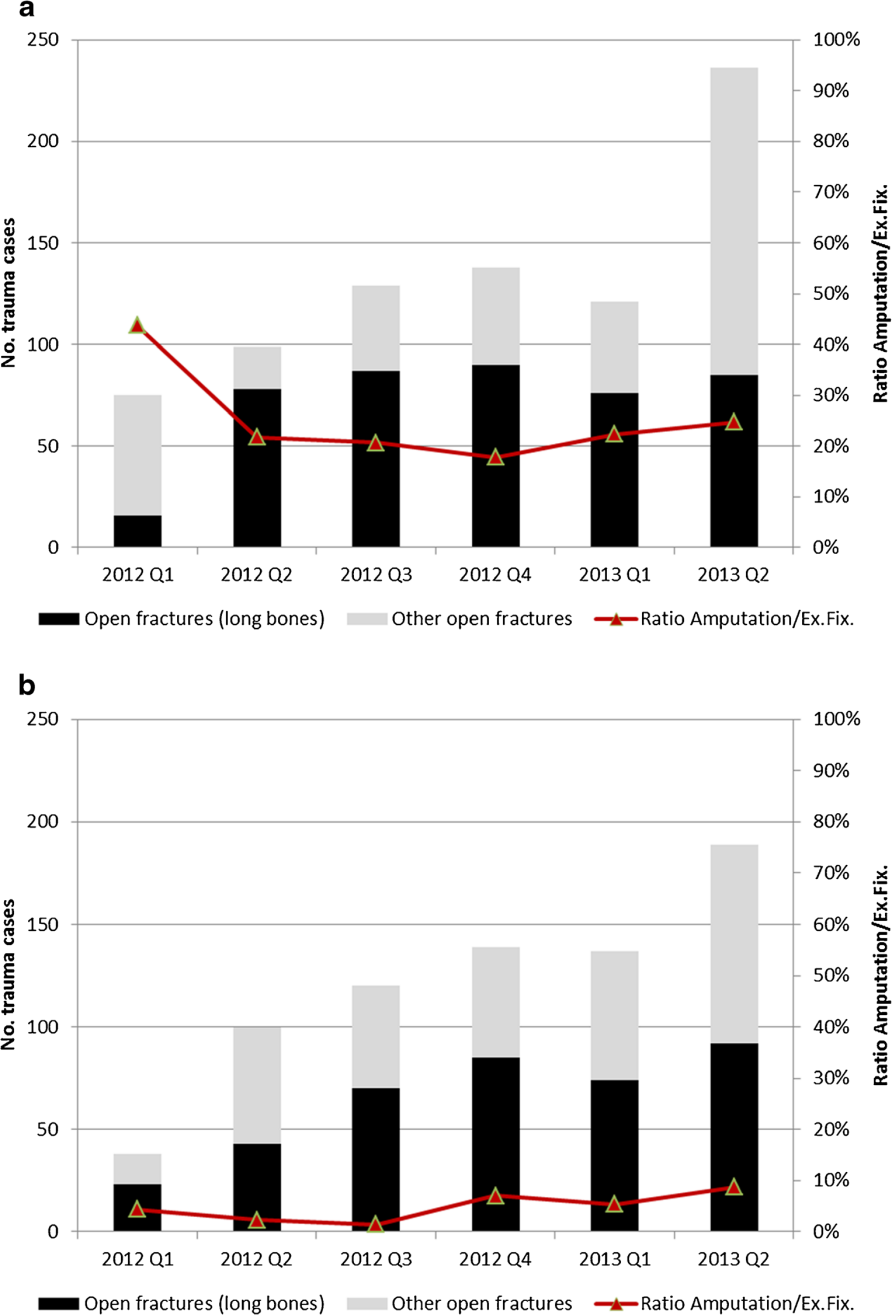
Type of open fracture cases and ratio of amputation versus external fixation among open fracture cases in the MSF-OCB trauma centres of Kunduz, Afghanistan (**a**) and Tabarre, Haiti (**b**), 2012–2013 (2nd quarter)

Over the study period, the emergency department of the Kunduz Trauma Centre in Afghanistan received a total of 798 open fractures; out of these, 168 (21 %) were the result of violence and 630 (79 %) the result of accidents. Over roughly the same period, the Tabarre Trauma Centre in Haiti received a total of 723 open fractures, 165 (23 %) of which were the result of violence and 558 (77 %) the result of accidents. The skeletal region of the fractures differed by the origin of the trauma, with violent trauma resulting in higher proportions of fractures of the radius/ulna, humerus and femur in both contexts (Fig. 4). A review of the interventions done for open fractures in long bones shows that the ratio of amputation to external fixation was relatively high in Afghanistan, starting at 50 % in the first quarter of 2012 and dropping to approximately 20 %, while it remained consistently low (<10 %) in Haiti (Fig. 5). Overall, 96 of 432 (22 %) open fractures of long bones in Afghanistan were treated by amputation, compared to 21 out of 387 (5 %) in Haiti.

## Discussion

Damage control orthopaedics under field conditions, in particular in LMIC, has always been a challenging balancing act between provision of quality care and the limited resources (both material and human resources) at hand. As a humanitarian organisation providing healthcare to individuals who often have no access to surgical care, MSF-OCB currently has three projects where orthopaedic surgery is performed, relying on national surgical staff who have different levels of surgical expertise, but use the same standard external fixator for the treatment of open fractures in long bones.

During the emergency response to the 2010 Haiti earthquake, the majority of interventions performed on open fracture cases were amputations, in particular in the early phase of the response. This may have been related to the emergency nature of the intervention; due to the overwhelming numbers of patients requiring immediate care, and the limited resources for external fixation (including availability of fixation material and challenges in ensuring adequate follow-up for patients with an external fixation), limbs were often considered unsalvageable under the programme conditions. Additionally, the first providers of surgical care in the post-earthquake response were general surgeons, who may not have been trained in specific orthopaedic procedures such as external fixation.

In long-term projects, the situation was different. In the conflict area of Masisi, DRC, the doctors on call in the emergency department of the General Referral Hospital were in general quick to decide to amputate on patients who arrived with open Gustilo type III fractures with massive soft tissue destruction. These doctors were non-specialists, as is most often the case in such contexts, but were honed by time and necessity in their respective fields. They were very adept in performing Caesarean sections, but were less adept in their routine work to the treatment options for orthopaedic cases. The steady decline in amputations over the years was achieved through training sessions in the field by expatriate orthopaedic surgeons who supervised and trained the medical doctors to perform basic and correct orthopaedic care, with an emphasis on the conservative and surgical options for specific fractures and dislocations. While the doctors may not have been particularly comfortable using the external fixation system, they were cognisant of the proper treatment and immobilization of open fractures.

In the specialised Kunduz Trauma Centre in Afghanistan, also a conflict setting, the national surgical staff was from the outset supported by expatriate orthopaedic surgeons, and the improvement of their skills over time could explain the relatively high uptake of external fixation techniques for the treatment of open fractures. In a similar trauma centre in Haiti, providing care to trauma patients in a non-conflict setting, the number of amputations was consistently low, i.e. since its inception, a limb salvage rate of 95 % was achieved. The staff in this centre was composed of musculoskeletal surgeons adept at using the external fixation system when deemed appropriate, which was likely a leading cause of these excellent results.

In summary, Médecins Sans Frontières trains medical doctors, surgeons and orthopaedic surgeons to provide patients with multiple options for quality care of open fractures, to improve treatment of open fractures, to save life and limb, and to prepare doctors for the eventuality of facing yet another mass casualty from a natural disaster. This review of the surgical activities in MSF-OCB projects performing orthopaedic care reveals that the introduction of and training on the proper utilization of external fixators reduced the amputation rate for open fractures and consequently increased the limb salvage rates.
